# Active Case Finding of Pulmonary Tuberculosis through Screening of Respiratory Symptomatics Using Sputum Microscopy: Is It Time to Change the Paradigm?

**DOI:** 10.1155/2013/312824

**Published:** 2013-02-28

**Authors:** Eva Carolina del Portillo-Mustieles, Rafael Laniado-Laborín

**Affiliations:** ^1^Clinica y Laboratorio de Tuberculosis, Hospital General de Tijuana, Avenida Centenario 10851, Zona Rio, Tijuana Baja California, CP 22320, Mexico; ^2^Facultad de Medicina y Psicología, Universidad Autónoma de Baja California, Mexico

## Abstract

*Background.* One of the main strategies for the early detection of pulmonary tuberculosis (PTB) is through the screening of individuals with symptoms compatible with PTB. Although this is programmatic strategy for active case finding, its yield is not well known. *Objective.* To determine the yield of pulmonary tuberculosis active case finding through the screening of respiratory symptomatic (RS) patients at a general hospital. *Methods.* RS patients were defined as subjects complaining of cough and/or sputum for a period of 2 or more weeks. Outpatients and their companions were approached while they waited in the outpatient care areas of the hospital to detect RS. Two samples from different days or 2 samples taken 2 hours apart on the same day were collected. *Results.* 122 RS patients were identified. Fifty-seven patients (46.7%) had at least one sputum sample analyzed. Three patients presented a positive smear and 2 were culture positive; neither had upper airway symptoms. None of the patients with productive cough and upper airway symptoms had a positive smear (*P* = 0.07). Only 19 (33.3%) returned to the laboratory to retrieve their results. *Conclusion.* Current strategy to screen RS patients based only on clinical data has a low compliance. Specific strategies to increase compliance (removal of barriers, incentives, etc.) should be implemented.

## 1. Introduction 

Tuberculosis (TB) represents one of the world's public health greatest challenges. One key factor for its control is to stop the chain of transmission in the community by diagnosing and treating cases as early as possible.

The planning and implementation of efficient tuberculosis control programs is crucial, and the strategies that compose them must be submitted to constant evaluation and refining. One of the main strategies for the early detection of pulmonary tuberculosis (PTB) is through the screening of individuals with symptoms compatible with PTB (in México for screening purposes defined as productive cough lasting more than two weeks), known also as respiratory symptomatic patients (RS).

It is estimated that in regions with high prevalence of PTB from 5 to 10% of the patients that seek outpatient medical services are RS, and, of those, from 1 to 5% are smear positive [1]. The most recent data from Mexico [2] shows a PTB incidence of 42.1/100,000 in the state of Baja California, the highest rate in the country; Tijuana the largest city in the state has an annual rate between 50 and 60 cases per 100,000. Most of the cases were detected passively at outpatient health services and hospitals; only 3.6% were detected by active screening of RS. 

Although the screening of RS is one of the control strategies for PTB in México, there is only one report on its yield. In the state of Tlaxcala, 6,748 subjects were interviewed and 245 (3.6%) were classified as RS, of which 17 (0.25%) were diagnosed with PTB [3]. 

On January 2012 the national TB control program gave instructions to the TB Clinic at the Tijuana General Hospital to carry out active case finding through the screening for RS at the hospital outpatient services (as opposite to passive detection of TB cases, where the patients seek medical care due to progressive respiratory symptoms). The objective of our study was to evaluate the efficacy of this screening strategy among outpatients of our hospital. 

## 2. Subjects and Methods 

The study was carried out from April to September 2012 in the outpatient care areas of the Tijuana General Hospital. This Mexican city has an estimated population of 2 million, and, as mentioned, the highest tuberculosis rate in the country. The hospital is a 180-bed facility that provides health care to the uninsured and poorest sector of the population. RS patients were defined as subjects complaining of productive cough for a period of 2 or more weeks [1].

Past experience of active search for RS in our hospital has shown a high percentage of patients that did not comply when asked to provide sputum samples or returned for their results. To increase our yield we implemented strategies such as providing verbal and written information regarding logistics and purpose of the sputum sample study, facilitating the sputum collection process by allowing the subjects to turn in 2 sputum samples in one day—as opposed to 3 samples from 3 consecutive days—and contacting those patients with positive results via telephone as soon as the results were obtained. 

Outpatients and their companions were approached while they waited in the outpatient care areas of the hospital and asked if someone was currently experiencing cough and/or sputum production that had lasted for 2 weeks or more weeks. If RS patients were detected they were asked to answer a brief questionnaire that included relevant demographic and clinical information. Subsequently they were given verbal and written indications on the requirements for adequate sputum collection, the number of samples needed, where to deliver the samples (TB laboratory location at the hospital, sputum reception hours, and laboratory direct telephone number), and the smear results reporting process. Initially patients were asked for 3 samples on 3 consecutive days, but since the majority of patients only brought from 1 or 2 samples to the laboratory, it was decided to change the collection to 2 samples from different days or 2 “on the spot” samples taken 2 hours apart on the same day, a strategy already validated in the literature [4, 5]. In the event that a spontaneous sputum sample could not be obtained, expectoration was induced with nebulized hypertonic saline solution. Samples were analyzed with the Ziehl-Neelsen technique on concentrated specimens [6]. 

If smear positive cases did not return within 3 days to pick up their results, they were contacted by telephone and referred to their community health center to initiate treatment. In smear positive cases an additional sample was collected for culture confirmation and drug susceptibility testing.

*Statistical Analysis*. Data analysis was done using the commercial statistical package SPSS 17.0 (SPSS Inc., Chicago, IL, USA), both for descriptive and inferential analysis. Continuous variables were analyzed with Student's *t*-test for independent samples and discrete variables using the Chi-square test (with Fisher's exact test for cells with <5 observations). Statistical significance was set as *P* < 0.05. 

## 3. Results 

One hundred twenty-two patients were included in the study. The mean age was 43.3 ± 15.3 years; 68% of the subjects were women. Three subjects (2.5%) were HIV positive. The frequencies of symptoms are presented in Table 1. 

Of the 122 subjects that agreed to have their sputum analyzed for TB, only 57 (46.7%) brought at least one sample to the tuberculosis laboratory. Laboratory technicians graded the samples from 20 patients (35.1%), as deficient in quality (mostly saliva). Only 19 patients (33.3% out of 57) returned to the laboratory to retrieve the results of their tests; they did so within an average of 9.63 ± 9.51 days after the scheduled date (range from 3 to 35 days). 

Three patients had an acid-fast-positive smear (2.45% of the total surveyed and 5.26% of those 57 that had at least one smear test done). However, only 2 of the smear positive patients had also a positive culture to confirm the diagnosis (one of them with a resistant strain to isoniazid, pyrazinamide, and streptomycin). The third smear positive case had a very low number of bacilli (2 in 200 fields), and the Lowenstein Jensen culture was negative after 9 weeks. None of the HIV-positive patients had a positive smear result. Nine patients (16.8%) were diabetics; none of them had a positive smear test for TB. Five patients that submitted samples (8.7%) reported alcoholism, 3 (5.2%) illegal substance abuse (1 with positive smears and culture), and 13 (22.8%) were smokers (2 had a positive smear and culture, one of them also used illegal substances; *P* = 0.12). 

Forty-one patients (71.9%) who complained of upper respiratory airway symptoms besides cough and sputum had at least one sputum sample analyzed; none of them was reported as positive. In contrast, 2 of the 16 patients that did not present upper airway symptoms were smear and culture positive (12.5%; *P* = 0.07). The patient with the paucibacillary smears and negative culture had a normal chest radiograph; it was decided not to start antituberculous treatment. On follow up a new set of smears and culture were negative; therefore, the initial smear was considered as a false positive. 

The patient with the drug resistant strain was the only patient with a history of PTB in the past; three subjects referred having had recent contact with a confirmed PTB case; however, none of them was smear positive. 

## 4. Discussion 

The screening for RS in this setting revealed that even though the TB laboratory is located within the hospital (avoiding the necessity to commute to another location), less than 50% of the subjects that agreed to participate provided sputum samples for analysis, and, of the ones who did, only a third returned to retrieve their results. In fact, it was necessary to locate via telephone 2 of the 3 smear positive patients since they had not returned to find out their result. The yield of our screening of RS was of 3.5% (2 patients with positive smears out 57 patients who provided samples), a similar percentage to that reported in the literature. In a report from Colombia [7] on RS screening at a hospital setting, the yield of positive smear patients was 3.7%; in that study, patients submitted an average of 1.6 samples, a number virtually identical to the one obtained in our study (1.7 samples per patient).

The yield of RS screening for TB could be increased if it was targeted on high-risk groups for TB. Screening for RS is usually carried out in outpatient health care establishments (community health centers, clinics, etc.) and, only in some cases, in the community by targeting high-risk groups for PTB [8, 9]. The yield of screening increases when the search is focused in high-risk populations such as prison populations, illegal drugs users, and homeless subjects [10]. Another reason is that respiratory symptoms are unspecific; 72% of our patients had upper respiratory symptoms besides productive cough and none of them had positive test. 

In the past, industrialized countries used mobile miniature X-ray units as a screening tool for active TBP cases, but the declination of TB rates and the cost of the procedure caused the practice to be abandoned [11]. Also, one of the inherent problems of this strategy was that it was applied in an indiscriminate manner, with no prior subject selection based upon clinical data or risk factors. The global reemergence of TB and the concentration of the cases in high-risk groups has offered the opportunity of bringing back radiographic screening of RS. The radiographic screening's feasibility has also increased due to the availability of modern digital techniques, with low-dose radiation at very low cost in mobile units. 

Radiographic screening has a low positive predictive value but an excellent negative predictive value (99.1%) [12]; RS patients could be screened initially with digital radiography and subjects with a normal chest would not be referred for sputum smear analysis (patients with HIV would be an exception since up to a fifth of HIV-infected persons with pulmonary TB have normal chest radiograph findings) [13]. It is possible that if the patient knows that they have an abnormal radiograph has they might be more compliant in the submission of sputum samples for tuberculosis analysis. A recent study [14], compared three PTB screening strategies: clinical screening based on suggestive symptoms (cough, hemoptysis, fever, nocturnal diaphoresis, and weight loss), chest radiography, and a combination of both. The combined screening method showed a sensitivity of 100%; logistic regression showed that radiographic abnormalities, cough, and weight loss were independent predictors for PTB diagnosis. 

## 5. Conclusions 

Yield of our current strategy to screen RS could be increased by improving compliance. The strategy must be modified by implementing some type of incentive for the patients to provide their sputum samples for analysis (food or transportation vouchers would be a good option for our patient population). Also a combination of screening tools including digital radiography [15], if available, would increase the yield and prove to be more cost efficient in the detection of active TB cases and reduce the number of false negative and false positive results.

## Figures and Tables

**Table 1:** Frequency of symptoms.

Symptoms	Number	%
Cough/sputum for ≥2 weeks	122	100
Fever	17	13.9
Night sweats	38	31.1
Hyporexia	15	12.3
Weight loss	21	17.2
*Upper airway allergy symptoms	89	73

*Conjunctivitis, rhinorrhea, nasal congestion, sore throat, and otalgia.

## References

[1] Farga V, Caminero J (2011). *Tuberculosis*.

[3] Vaca-Marín MA, Tlacahuac-Cholula C, Olvera-Castillo R (1999). Pulmonary Tuberculosis among respiratory symptomatics detected in SSA health units in the state of Tlaxcala, Mexico. *Revista del Instituto Nacional de Enfermedades Respiratorias Mexico*.

[4] Mase SR, Ramsay A, Ng V (2007). Yield of serial sputum specimen examinations in the diagnosis of pulmonary tuberculosis: a systematic review. *International Journal of Tuberculosis and Lung Disease*.

[5] Hirao S, Yassin MA, Khamofu HG (2007). Same-day smears in the diagnosis of tuberculosis. *Tropical Medicine and International Health*.

[6] American Thoracic Society (2000). Diagnostic standards and classification of tuberculosis in adults and children. *American Journal of Respiratory and Critical Care Medicine*.

[7] Henao-Riveros SC, Sierra-Parada CR, Sánchez-Morales EA, Saavedra-Rodríguez A (2007). Búsqueda de tuberculosis en pacientes sintomáticos respiratorios en cuatro hospitales de Bogotá DC. *Revista Española de de Salud Pública*.

[8] Programa de Accion Tuberculosis, Secretaria de salud—Subsecretaria de prevencion y proteccion de la salud.

[9] Manuales de Capacitacion para el Manejo de la Tuberculosis. Módulo 2: Detección de casos tuberculosis.

[10] Julia Inés EM, William MO, Julio César GL (2003). Active research of respiratory symptomatic patients in high-risk population for tuberculosis. *Revista Facultad Nacional de Salud Pública*.

[11] Iademarco MF, Grady J O, Lönnroth K (2012). Chest radiography for tuberculosis screening is back on the agenda. *The International Journal of Tuberculosis and Lung Disease*.

[12] Story A, Aldridge RW, Abubakar I (2012). Active case finding for pulmonary tuberculosis using mobile digital chest radiography: an observational study. *The International Journal of Tuberculosis and Lung Disease*.

[13] Sterling TR, Pham PA, Chaisson RE (2010). HIV infection-related tuberculosis: clinical manifestations and treatment. *Clinical Infectious Diseases*.

[14] van’t Hoog AH, Meme HK, Laserson KF (2012). Screening strategies for tuberculosis prevalence surveys: the value of chest radiography and symptoms. *PLoS ONE*.

[15] Coulborn MR, Panunzi I, Spijker S (2012). Feasibility of using teleradiography to improve tuberculosis screening and case management in a district hospital in Malawi. *Bulletin of the World Health Organization*.

